# Genetic Variant rs401681 at 5p15.33 Modifies Susceptibility to Lung Cancer but Not Esophageal Squamous Cell Carcinoma

**DOI:** 10.1371/journal.pone.0084277

**Published:** 2013-12-30

**Authors:** Man Jiang, Haijian Wu, Chengyong Qin

**Affiliations:** 1 Department of Gastroenterology, Provincial Hospital Affiliated to Shandong University, Jinan, Shandong Province, China; 2 Department of Radiation Oncology, Qilu Hospital Affiliated to Shandong University, Jinan, Shandong Province, China; Virginia Commonwealth University, United States of America

## Abstract

**Background:**

The human 5p15.33 locus contains two well-known genes, the telomerase reverse transcriptase (*TERT*) and cleft lip and palate transmembrane 1-like (*CLPTM1L*) genes, which have been implicated in carcinogenesis. A common sequence variant, rs401681, located in an intronic region of *CLPTM1L*, has been reported to be associated with lung cancer risk based on genome-wide association study. However, subsequent replication studies in diverse populations have yielded inconsistent results. In addition, genetic variants at 5p15.33, including rs401681, have been shown to be involved in the susceptibility to multiple malignancies. Nevertheless, the role of these *TERT-CLPTM1L* variants in the etiology of esophageal squamous cell carcinoma (ESCC) remains unknown.

**Methods:**

We genotyped the rs401681 polymorphism using TaqMan methodology and analyzed its association with the risk of lung cancer and ESCC in a case–control study of 1,479 cancer patients (726 with lung cancer and 753 with ESCC) and 860 healthy individuals.

**Results:**

Logistic regression analyses revealed that rs401681 T genotypes were associated with a significantly decreased risk of lung cancer (CT vs. CC: adjusted OR = 0.782, 95% CI = 0.625–0.978, *P* = 0.031; CT/TT vs. CC: adjusted OR = 0.786; 95% CI = 0.635–0.972, *P* = 0.026). Stratification analysis by histology type indicated that rs401681 T genotypes were associated with a significantly reduced risk of both adenocarcinoma and squamous cell carcinoma. Furthermore, no significant association was observed between rs401681 and the risk of ESCC (CT vs. CC: adjusted OR = 0.910, 95% CI = 0.734–1.129, *P* = 0.392; TT vs. CC: adjusted OR = 0.897, 95%CI = 0.624–1.290, *P* = 0.558; CT/TT vs. CC: adjusted OR = 0.908, 95% CI = 0.740–1.114, *P* = 0.355).

**Conclusions:**

Our findings provide further evidence supporting rs401681 as a genetic variant associated with the risk of lung cancer. In addition, we investigated the correlation between the rs401681 variant and the risk of ESCC in a Han Chinese population, and our results suggest that this genetic variant may not be involved in ESCC risk.

## Introduction

Lung cancer has been the most common cancer in the world for several decades as well as the most common cause of death from cancer. In addition, esophageal cancer is the eighth most common cancer worldwide, with 482,000 new cases estimated in 2008, and represents the sixth most common cause of death from cancer, being responsible for 407,000 deaths [1]–[2]. The mortality rates from lung cancer and ESCC are particularly high in China [3]–[4].

Recently, genome-wide association studies (GWASs) have shown that common *TERT-CLPTM1L* variants at 5p15.33 may influence the risk of developing lung cancer as well as other types of cancer [5]–[11]. Notably, by pooling the available GWAS data on lung cancer, Timofeeva et al. [12] identified common susceptibility loci at 5p15 and demonstrated histology-specific effects of 5p15 loci. Both *TERT* and *CLPTM1L* are attractive candidate genes, as they have both been implicated in carcinogenesis. *TERT* encodes the catalytic subunit of telomerase, an enzyme that maintains telomere ends by adding the telomere repeat TTAGGG. Telomeres are the protein-bound DNA repeat structures at the ends of chromosomes and are important in maintaining genomic stability [13]–[15]. Another potential candidate causal gene in the 5p15.33 region is *CLPTM1L*, which has been reported to be involved in the cellular response to genotoxic stress and cisplatin resistance [16]. In addition, over-expression of *CLPTM1L* has been observed in several types of cancer, including lung cancer [17]–[20].

The rs401681 polymorphism, located in an intronic region of *CLPTM1L*, has been reported to be associated with lung cancer risk based on genome-wide association study [5]. Nevertheless, subsequent replication studies in diverse populations have yielded inconsistent results. Myneni et al. [21] and Ke et al. [22] found that rs401681 was significantly associated with lung cancer risk in the Asian population, whereas another study examining 501 lung cancer cases and 576 cancer-free controls detected no association between this genetic variant and risk of lung cancer in a population of the same ethnicity [23]. Additionally, a study in a Korean population found that rs401681 was associated with a significantly decreased risk of lung cancer under a dominant model for the variant allele [24]. However, no significant association between this genetic variant and lung cancer risk was observed in Norwegian Caucasians [25]. Different study designs and sample sizes as well as histological heterogeneity may contribute to the discrepancies observed in these studies. Furthermore, given the differences in genetic background and linkage disequilibrium patterns across populations, it is expected that independent studies conducted in diverse populations will shed further light on the role of this genetic variant in cancer risk.

In most cases, the common genetic variants that have been associated with cancer risk seem to be specific to a particular cancer type. However, single-nucleotide polymorphisms (SNPs) in the *TERT-CLPTM1L* region, including rs401681, have shown possible associations in multiple cancers [26]–[28]. Recently, rs401681[C] was reported to be associated with an increased risk of basal cell carcinoma and lung, urinary bladder, prostate and cervix cancers. Conversely, rs401681[C] appears to confer protection against cutaneous melanoma [6], [7]. Moreover, a recent GWAS demonstrated that rs401681 may modify individual susceptibility to pancreatic cancer [8]. The rs401681 polymorphism has been widely studied in different ethnicities and cancer types. However, the role of this genetic variant in ESCC susceptibility is still unknown. Here, we attempted to address these issues by conducting a case–control study in a Han Chinese population.

## Materials and Methods

### Study Subjects

This case-control study included 726 patients with lung cancer, 753 patients with esophageal cancer and 860 healthy controls. All of the subjects in this study were genetically unrelated ethnic Han Chinese individuals from Shandong Province in North China. The cases with histologically confirmed primary lung or esophageal cancer were recruited from 2011 to 2013 at Qilu Hospital and the Provincial Hospital Affiliated with Shandong University (Jinan, China). The histological type of the tumors was diagnosed on the basis of biopsies or resected specimens. The esophageal carcinomas were all squamous cell carcinomas. Patients with primary cancer outside the lung and the esophagus and with cancer of unknown primary origin were excluded. The healthy participants, who presented no history or diagnosis of cancer or genetic disease, were recruited from individuals who visited the same hospitals for a routine check-up during the same period. Patients or controls who had recently (in the last 6 months) received blood transfusions were excluded. Cases and controls were frequency matched by age (±5 years) and sex. All participants were given an explanation of the study, and written informed consent was obtained from each subject. This study was approved by the Ethics Committees of the Provincial Hospital Affiliated with Shandong University.

### Data Collection

A structured questionnaire was completed for each case and control by a trained interviewer to obtain demographic data (e.g., age, sex) and information on related risk factors (including tobacco smoking and alcohol consumption). Individuals who had smoked one cigarette per day for over 1 year were considered smokers. Subjects were considered alcohol drinkers if they drank at least once per week for over 1 year.

### DNA Extraction and Genotyping

A venous blood sample was collected from each subject, and genomic DNA was extracted within 1 week after sampling using the RelaxGene Blood DNA System (Tiangen Biotech (Beijing) Co., Ltd.) according to the manufacturer’s protocol. The quality and quantity of DNA were checked using GeneQuant Pro (Amersham Biosciences). The rs401681 polymorphism was genotyped using TaqMan methodology in 96-well plates and read with Sequence Detection Software (SDS, version 1.4) using the Applied Biosystems (ABI) 7500 Real-Time PCR System. Genotyping was performed without knowledge of the subjects’ case or control status. The genotyping assays were randomly repeated for 12% of the samples, and the results were 100% concordant.

### Statistical Analysis

Pearson’s χ^2^ test was used to examine the differences in the distributions of category variables, including demographic characteristics, selected variables, and genotype/allele frequencies, between the cases and controls. Hardy–Weinberg equilibrium was tested using a goodness-of-fit χ^2^ test to compare the observed genotype frequencies to the expected frequencies among the control subjects. The associations between the rs401681 polymorphism and cancer risk were estimated by computing the odds ratios (ORs) and their 95% confidence intervals (CIs) through logistic regression analyses for crude ORs and adjusted ORs when adjusting for age, sex and smoking and drinking status. Statistical analyses were carried out using SPSS (version 19) and Stata (version 12.0), and a *P*-value of <0.05 was set as the criterion for statistical significance.

## Results

### Subject Characteristics

A total of 1,479 cancer patients (726 with lung cancer and 753 with ESCC) and 860 healthy controls were included in the analysis. The demographic distributions of the lung cancer and ESCC patients and healthy controls are shown in Table 1. The age and sex distributions were not significantly different between the cases and controls. However, there were more ever smokers among the lung cancer and ESCC patients (66.4 and 59.1%, respectively) than in the control group (34.7%; *P*<0.001 for both). In addition, the proportion of alcohol drinkers in both the lung cancer and ESCC patient groups was significantly different from that in healthy controls. Therefore, these variables were further adjusted for in subsequent multivariate logistic regression analyses. Within the lung cancer group, patients with squamous cell carcinoma, adenocarcinoma, small-cell carcinoma, and other carcinomas accounted for 34.6%, 37.7%, 19.4%, and 8.3% of the subjects, respectively. The esophageal carcinomas were all squamous cell carcinomas.

**Table 1:** Characteristics of lung cancer and esophageal squamous cell carcinoma (ESCC) patients and healthy controls.

Variables		Controls (n = 860)		Lung cancer (n = 726)			ESCC(n = 753)		
		n	%	n	%	*P*-value*	n	%	*P*-value*
Age (years)									
	≤60	446	51.9	389	53.6	0.494	381	50.6	0.613
	>60	414	48.1	337	46.4		372	49.4	
Sex									
	Male	703	81.7	579	79.8	0.315	604	80.2	0.434
	Female	157	18.3	147	20.2		149	19.8	
Smoking status									
	Ever	298	34.7	482	66.4	<0.001	445	59.1	<0.001
	Never	562	65.3	244	33.6		308	40.9	
Alcohol status									
	Ever	314	36.5	372	51.2	<0.001	433	57.5	<0.001
	Never	546	63.5	354	48.8		320	42.5	
Histology									
	SQC			251	34.6		753	100	
	Adenocarcinoma			274	37.7				
	SCC			141	19.4				
	Other carcinomas			60	8.3				

Abbreviations: SQC, squamous cell carcinoma; SCC, small-cell carcinoma.

Two-sided χ^2^ test for categorical variables.

### Association between rs401681 and the Risk of Lung Cancer and ESCC

The genotype and allele frequencies of the rs401681 polymorphism in the lung cancer and ESCC patients and healthy controls are presented in Table 2. Among the 860 control subjects, the genotype frequencies were 45.9% CC, 44.0% CT and 10.1% TT, and the allele frequencies were 67.9% C and 32.1% T. The observed genotype frequencies among the control subjects complied with Hardy-Weinberg equilibrium. Overall, the genotype distribution in the lung cancer group was not significantly different from that in the healthy controls (*P* = 0.121). However, a significant difference in the combined genotype (CT+TT) frequency was observed between patients with lung cancer and control subjects (*P* = 0.040).

**Table 2:** Genotype and allele frequencies of the rs401681 polymorphism in lung cancer and esophageal squamous cell carcinoma (ESCC) patients and healthy controls.

Genotype/Allele		Controls* (n = 860)		Lung cancer (n = 726)			ESCC(n = 753)		
		n	%	n	%	*P*-value	n	%	*P*-value
Genotype									
	CC	395	45.9	371	51.1		360	47.8	
	CT	378	44.0	289	39.8		321	42.6	
	TT	87	10.1	66	9.1	0.121^a^	72	9.6	0.744^a^
	CT+TT	465	54.1	355	48.9	0.040^b^	393	52.2	0.451^b^
Allele									
	C	1168	67.9	1031	71.0		1041	69.1	
	T	552	32.1	421	29.0	0.059^c^	465	30.9	0.458^c^

^a^ Two-sided χ^2^ test of the difference in the genotype frequency distribution between cases and controls.

^b^ Two-sided χ^2^ test of the distribution of combined genotypes (CT+TT).

cTwo-sided χ^2^ test of the allele distribution.

The observed genotype frequency among the control subjects conformed to Hardy–Weinberg equilibrium (p^2^+2pq+q^2^ = 1)(*P* = 0.805).

**Figure 1:**
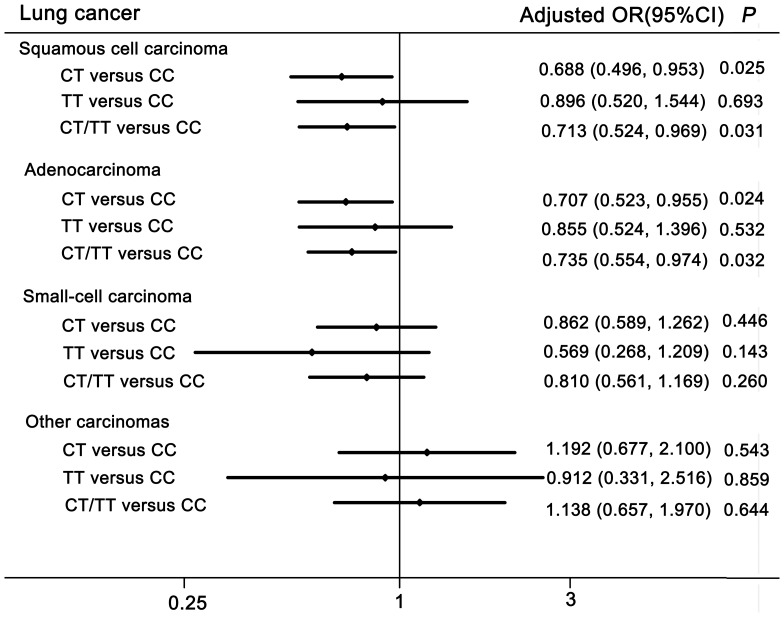
Forest plot of lung cancer risk associated with the rs401681 polymorphism. Stratification analysis by histology type revealed that rs401681 T genotypes were associated with a significantly reduced risk of both adenocarcinoma and squamous cell carcinoma.

As shown in Table 3, logistic regression analyses revealed that rs401681 T genotypes were associated with a significantly decreased risk of lung cancer (CT vs. CC: adjusted OR = 0.782, 95% CI = 0.625–0.978, *P* = 0.031; CT/TT vs. CC: adjusted OR = 0.786; 95% CI = 0.635–0.972, *P* = 0.026). Moreover, the rs401681 T allele was borderline significantly associated with a lower risk of lung cancer. We further evaluated the association between rs401681 and lung cancer risk stratified by histology type. As shown in Figure 1, stratification analysis by histology type indicated that rs401681 T genotypes were associated with a significantly reduced risk of both adenocarcinoma and squamous cell carcinoma, but not with small-cell carcinoma.

**Table 3:** Association between the rs401681 polymorphism and the susceptibility to lung cancer and esophageal squamous cell carcinoma (ESCC).

	Genotype/Allele	Crude OR and *P* value		Adjusted OR and *P* value	
		OR(95%CI)^a^	*P* a	OR(95%CI)^b^	*P* b
Lung cancer					
	CC	Reference		Reference	
	CT	0.814 (0.661, 1.003)	0.053	0.782 (0.625,0.978)	0.031
	TT	0.808 (0.569, 1.146)	0.232	0.807 (0.552, 1.179)	0.267
	DOM	0.813 (0.667, 0.991)	0.040	0.786 (0.635, 0.972)	0.026
	REC	0.889 (0.635, 1.244)	0.491	0.906 (0.632, 1.299)	0.592
	ADD	0.864 (0.742, 1.006)	0.059	0.851 (0.723, 1.001)	0.052
**ESCC**					
	**CC**	**Reference**		**Reference**	
	CT	0.932 (0.758, 1.145)	0.502	0.910 (0.734, 1.129)	0.392
	TT	0.908 (0.644, 1.280)	0.582	0.897 (0.624, 1.290)	0.558
	DOM	0.927 (0.762, 1.128)	0.451	0.908 (0.740, 1.114)	0.355
	REC	0.939 (0.676, 1.305)	0.709	0.946 (0.671, 1.335)	0.754
	ADD	0.945 (0.814, 1.097)	0.458	0.935 (0.800, 1.093)	0.398

Abbreviations: OR, odds ratio; CI, confidence interval; DOM, dominant model (CT/TT vs. CC); REC, recessive model (TT vs. CC/CT); ADD, additive model (2*TT+CT vs. 2*CC+CT).

^a^ Crude ORs and *P* values were estimated in a logistic regression model without any adjustment.

^b^ Adjusted for age, sex and smoking and alcohol status.

For the patients with esophageal carcinoma, the genotype frequencies of the rs401681 variant were not significantly different between the cases and controls. In addition, the rs401681 T allele was less frequent among cases than among controls, though this difference was not statistically significant (Table 2). Following adjustment for age, sex and smoking and drinking status in a multivariate logistic regression analysis, no significant association was observed between rs401681 and the susceptibility to esophageal carcinoma (CT vs. CC: adjusted OR = 0.910, 95%CI = 0.734–1.129; TT vs. CC: adjusted OR = 0.897, 95%CI = 0.624–1.290; CT/TT vs. CC: adjusted OR = 0.908, 95%CI = 0.740–1.114) (Table 3).

## Discussion

Telomere biology has been implicated in the pathogenesis of a variety of lung diseases [29]. A previous study indicated that telomere dysfunction may act as both a potent tumor suppressor and promoter in the lung epithelial compartment, depending on the status of the telomere dysfunction-induced checkpoint [30]. In advanced NSCLC, a high pretreatment level of circulating TERT may serve as an independent poor prognostic marker for overall survival [31]. Telomere biology plays a critical and complex role in the initiation and progression of cancer [32], [33]. The exact functional relevance of the rs401681 polymorphism regarding its association with lung cancer currently remains unclear, but some previous studies provide a link to this association [6], [25]. This variant may be in strong linkage disequilibrium (LD) with other potential functional or causal SNPs contributing to the risk of lung cancer. For example, *CLPTM1L* rs402710, which is in strong LD with rs401681, has been reported to be associated with significantly higher levels of bulky aromatic/hydrophobic DNA adducts in lung tissue adjacent to tumors [25]. Moreover, rs401681[C] has been shown to be associated with shorter telomeres [6]. Consistent with previous observations from a pooled analysis [26], the results of our case–control study suggested that rs401681 T genotypes were associated with a significantly reduced risk of both lung adenocarcinoma and squamous cell carcinoma. A large pooled analysis conducted by Timofeeva et al. confirmed that two independent susceptibility variants at 5p15.33 (*TERT* rs2736100 and *CLPTM1L* rs401681) act as determinants of lung cancer risk, differentially impacting lung cancer histology. The risk associated with rs2736100 is largely confined to adenocarcinoma. However, rs401681 influences the risk of all lung cancer histologies, but its strongest effect is on squamous cell carcinoma [12].

A recent meta-analysis by cancer type demonstrated that rs401681 [T] carriers show a modestly increased risk of pancreatic carcinoma and skin melanoma. In contrast, a modest reduction of risk was observed for bladder, lung and prostate cancer as well as basal cell and skin squamous cell carcinomas [26]. In the present case-control study, the rs401681 T allele was less frequent among the esophageal cancer cases than the controls, though this difference was not statistically significant. Following adjustment for age, sex and smoking and drinking status in a multivariate logistic regression analysis, no significant association was found between rs401681 and the susceptibility to ESCC. Several studies have reported an association between short telomeres and an increased risk of esophageal cancer [34]–[38]. A previous genome-wide association study demonstrated that four SNPs (rs621559 on 1p34.2, rs398652 on 14q21, rs6028466 on 20q11.22 and rs654128 on 6q22.1) were associated with leukocyte telomere length in Caucasian populations [39]. In a subsequent study of 1550 ESCC patients and 1620 controls in China, Shi et al. [40] found that both rs621559 and rs398652 were significantly associated with ESCC risk in additive, recessive or dominant genetic models. However, in the present study, we detected no association between rs401681 and the risk of ESCC, although rs401681[C] has been reported to be associated with shorter telomeres with nominal significance [6].

Several limitations of our study should be discussed. First, selection bias may not be avoidable. However, the cases and healthy control subjects were recruited from the same area and matched for age and sex. Second, the sample size in this study was not sufficiently large to draw a firm conclusion, and the statistical power of the study may be limited. Third, we cannot exclude the potential roles of other functional SNPs or tagging SNPs for *TERT-CLPTM1L* that we did not include in the present study of ESCC risk. Finally, we incorporated the major confounding variables in our analysis. However, other detailed risk factors were not included in our logistic regression models because of incomplete or missing information.

In summary, our findings suggested that rs401681 may modify susceptibility to lung cancer but not ESCC. Although no significant association was found between this genetic variant and susceptibility to ESCC, more variants at the *TERT-CLPTM1L* locus need to be investigated to further evaluate the role of this genomic region in esophageal tumorigenesis.
